# Mutation of PTEN: Loss and Likelihood of Being a Non-responder to Trastuzumab in a Sample of Iraqi Her2+ Breast Cancer Patients

**DOI:** 10.7759/cureus.54765

**Published:** 2024-02-23

**Authors:** Alyaa H Hammadi, Shatha H Ali

**Affiliations:** 1 Department of Clinical Pharmacy, College of Pharmacy, University of Baghdad, Baghdad, IRQ; 2 Department of Clinical Laboratory Science, College of Pharmacy, University of Baghdad, Baghdad, IRQ

**Keywords:** phosphatase and tensing homolog, creatine kinase-mb, troponin, phosphoinositide 3-kinase response, trastuzumab, breast cancer

## Abstract

Introduction: PTEN controls upstream PI3K relatives, such as AKT. PTEN gene mutations have been documented to affect outcomes in main or distant malignancies, including breast cancer (BC). PTEN gene deletions are common in a variety of human cancers. A key factor in the response to this kind of therapy is genetic diversity. The purpose of this research is to determine whether a PTEN loss mutation influences a patient's propensity to not respond to trastuzumab (TRS) in cases of Her2+ BC.

Methods: Diwaniya Teaching Hospital's oncology ward provided 60 patients with Her2+ BC who had been on TRS for at least 12 months for this study. Patients were split in half using the RECIST criteria for evaluating responses to therapy in solid tumors: responders and non-responders. A PTEN polyclonal primary antibody was used for the detection of PTEN in breast tissue in the current study.

Results: This research employs a rating system based on eight specimens (26.67%) among non-responsive women who demonstrated PTEN loss compared with one specimen (3.33%) among responsive women. Statistically, PTEN loss varied significantly between the responsive and non-responsive groups. Loss of PTEN was also not linked to shifts in creatine kinase-myocardial band (CK-MB), troponin T (TnT), or any other biomarker, or troponin I (Tn1) at baseline or after 12 months of TRS therapy.

These results give us important information about how PTEN deletion mutations might work as a predictor for TRS response in women with Her2+ BC.

## Introduction

The most typical malignancy in Iraqi women is breast cancer (BC) [1]. BC is thought to be the primary cause of cancer death in women and accounts for around 25% of all new cancer cases identified in women worldwide [2-3]. The most significant health issue, cancer, tends to acquire drug resistance, which increases attempts to find new protocols for treating cancer [4]. Trastuzumab (TRS) is a humanized recombinant antibody that targets HER2. It was first approved for use in 1998 as a first-line treatment for HER2+ cancers that keep coming back [5]. When a medication was introduced to chemotherapy regimens for women with HER2+ BC, they demonstrated a significant improvement in their average lifespan [5]. Invasion and metastasis of BC cells are a multi-gene, multi-stage process that starts with oncogene mutations and ends with tumor suppressor genes being turned off [6,7]. It was found that the tumor suppressor gene phosphatase and tensin homolog (PTEN) works by blocking the PI3K/AKT pathway.

The cell cycle, energy metabolism, genomic stability, adhesion, migration, metastasis, and apoptosis are just some of the many cellular activities that PTEN is involved in. Previous studies have shown a critical role for PTEN mutation and functional loss in cancer initiation, progression, and metastasis [8]. Several studies have demonstrated that PTEN abnormalities in BC cells are primarily brought on by mutations, chromosome 10 deletions, aberrant methylation of the promoter DNA, and the subsequent loss of PTEN protein expression or function [9,10]. About 40%-50% of BC patients have been reported to have PTEN loss of heterozygosity, and 5%-10% of BC patients have PTEN mutations, the majority of which are frameshift mutations that impair PTEN function. The primary causes of PTEN inactivation are somatic mutations, epigenetic suppression by promoter methylation leading to mutations in the PTEN gene, mutations like missense and nonsense, as well as deletions of single or multiple copies of an entire gene, which lead to PTEN protein degradation and post-translational alterations [9,10]. Both prognostic and predictive settings have been investigated in BC regarding the clinical actionability of PTEN status. Patients with HER2+ BCs who had a poor response to TRS therapy showed changes in PTEN and the serine/threonine kinase AKT isoforms. Additionally, evaluation of PTEN expression has been suggested as an additional biomarker for determining BC's mismatch repair status.

This could help select patients, such as those who have tumors that express the hormone receptor (HR), who are eligible for immune-checkpoint blockade [11]. The Breast Cancer International Research Group 006 (BCIRG-006) trial, which was the third part of the study, showed that PTEN loss in people with HER2+ BC is linked to a worse prognosis but not to TRS resistance [12]. A meta-analysis of 27 studies comprising 10231 BCs offered more evidence that PTEN depletion may be a predictor of aggressive behavior. However, new clinical and translational research has failed to discover a link between PTEN status and health outcomes for patients. Therefore, it is unclear whether PTEN testing is consistent in clinical practice for patients with BC [13]. The goal of this study was to find out if having PTEN loss might change the chance of not responding to TRS. This study built on earlier research that looked at this possibility in Iraqi HER2+ BC patients.

## Materials and methods

The investigation described in this research article took place between October 12, 2021, and August 8, 2022, and was a large-scale observational cross-sectional study. Sixty women from Iraq who had been diagnosed with HER2+ BC and whose tumors had been staged using the modified American College of Oncology (ACO) response assessment criteria in solid tumors (RECIST) from 2010 were included in the research [14]. The participants were from the Oncology Department of Diwaniya Teaching Hospital in Diwaniya, Iraq. This unit provides assistance to citizens in rural, urban, and inner-city areas throughout a wide variety of governorates in Iraq. On October 7, 2021, the Rheumatology Medical Department at the Baghdad Teaching Hospital and the College of Pharmacy at the University of Baghdad granted an ethical clearance (RECAUBCP7102021A). In addition, everyone who took part gave their informed permission in writing. Sixty-nine patients who satisfied the following criteria for Her2+ BC were included in the research and treated with trastuzumab as a single treatment. However, only 63 patients volunteered to participate, and only 60 patients met all of the criteria.

Women with a biopsy-proven diagnosis of HER2+ BC were eligible for participation. Trastuzumab was administered to patients after 12 months of observation of normal bone marrow, liver, kidney, and heart function [15]. In a clinical setting, via endoscopy or using straightforward imaging techniques in radiology (such as plain film x-ray, CT, US, or MRI), patients also needed to have an intravenous infusion of trastuzumab at 8 mg/kg over the course of 90 minutes. Afterward, 6 mg/kg was intravenously infused over 30-90 minutes every three weeks for at least 12 months before enrollment, with no dose skipping.

Patients were not eligible if they had a history of cardiac disease, had received radiotherapy within the past 12 months, had received trastuzumab within the past 12 months, or had infections such as tuberculosis (TB), human immunodeficiency virus (HIV), or non-resolving active bacterial infections.

Patients were split in half using the response assessment criteria in solid tumors (RECIST) as the dividing line, after being on trastuzumab for at least a year [12]. When all tumors, both visible and detectable by imaging techniques, have disappeared, we say that the patient has achieved a complete response (CR). The presence of no new metastatic lesions and a reduction of at least 30% in the maximal diameter of existing metastatic lesions constitute a partial response (PR). The absence of new lesions and a decrease in the size of existing ones by at least 20% characterize stable disease (SD). A CR, PR, or SD at 12 months was considered a clinical benefit. In this study, patients who satisfied these criteria were considered "responders." Those patients who had disease progression (PD) and had either new lesions appear or the size of their existing lesions grow by more than 20% were considered “non-responders.” Based on the responses, we then divided the patients into two categories. Thirty patients with HER2+ BC who had a therapeutic response to Group B of the trastuzumab trial comprised 30 patients with HER2+ BC who did not show a response to trastuzumab.

Demographic information (age, weight, and length of sickness) was gathered via direct interviews with patients using a patient information system developed for the purpose of this study. By dividing each respondent's kilogram weight by their square meter height, we were able to calculate their body mass index (BMI) [16]. Each patient had five milliliters of blood drawn from a vein in their forearm. After the blood was drawn, it was placed in a tube containing ethylene diamine tetraacetic acid (EDTA) for DNA isolation. Meanwhile, the remaining 3 mL of blood were centrifuged at 4000 rpm in a gel tube for 10 minutes. Once we had finished collecting samples, we transferred the leftover serum to an Eppendorf tube and stored it in the freezer. Then, cardiac troponin I (Tn1), troponin T (TnT), and creatine kinase-myocardial band (CK-MB) were measured using the enzyme-linked immunosorbent assay (ELIZA) technique. The snulong ELIZA kit (China Catalog Nos. CSB-SL0536hu, CSB-Sl1747hu, and CSB-Sl1746hu) was used to check the levels of cardiac Tn1, TnT, and CK-MB in the blood. The method of quantitative sandwich enzyme immunoassay is used in this test [17,18].

The Dako EnVision detection immune histochemistry kit (Envision FLEX, Dako, K8000, Denmark) was used for immunohistochemistry as per the manufacturer's instructions.

In the present investigation, PTEN in breast tissue was detected using a polyclonal primary antibody (Polyclonal Rabbit Antibody: E-AB-19312 from Elabscience in Canada). Antibody diluent (EnVision FLEX Antibody Diluent, Dako, K8006, Denmark) was used to dilute a PTEN polyclonal primary antibody to 1 g/mL. The analysis was carried out using SPSS for Windows 26.0 (SPSS Inc., Chicago, IL, USA). We employed measures of central tendency, the mean and the standard deviation, to characterize continuous variables. Numerical and percentage representations of discrete variables were provided. A direct count report was done on allele and genotype frequencies and percentages. The mean difference of two independent samples was tested using the independent T-test. The chi-square test and the Fischer exact test were applied to study the link between two category variables. The impact of the genotypes on the likelihood of responding was calculated using a binary logistic regression analysis. Pearson's correlation coefficient (r) was used to examine the association between two variables. If the likelihood was less than 5%, then it was significant; if it was between 0.01 and 0.001, then it was very significant.

## Results

Table 1 provides a brief overview of the demographics of the test populations. Patients were paired in this trial. Clinically, however, for smoking status, there was a statistically significant split between those who responded and those who did not (P=0.04). After using TRS for a year, significant differences in CK-MB, TnT, and Tn1 levels were also found between responders and non-responders.

**Table 1:** Demographic data and clinical characteristic parameters of the study groups p<0.05 is considered significant. N, number of responders; BMI, body mass index; CK-MB, creatine kinase-myocardial band; TnT, troponin T; Tn1, troponin I

Variables		Responder group	Non-Responder group	P-value
		N=30	N=30	
Age (yrs)		51.86±8.148	48.76±8.601	0.1
BMI (Kg/m^2^)		63.37±8.277	63.20±9.297	0.1
Nonsmoker	Smoking status (N%)	5 (16.7%)	12 (40.0%)	0.04
Smoker		25 (83.3%)	18 (60.0%)	
Baseline	CK-MB	10.912±2.404	10.989±2.370	0.9
After 12 months	ng/mL	12.012±2.419	11.458±2.075	0.3
Baseline	TnT	158.696±185.82	134.652±26.429	0.4
After 12 months	Pg/mL	130.55±32.381	149.590±38.88	0.04
Baseline	Tn1	626.66±169.029	732.489±195.09	0.2
After 12 months	Pg/mL	671.177±151.33	1008.93±150.66	0.0001

**Figure 1:**
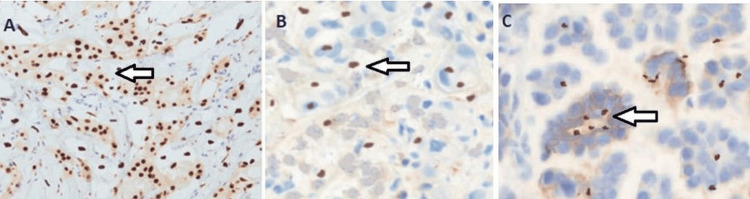
Representative pictures of immunostaining of PTEN protein expression in human breast tissue samples (A) Normal breast tissue showing strong PTEN expression, (B) BC tissue with negative PTEN expression, and (C) BC tissue with low PTEN protein expression. PTEN, phosphatase and tensin homolog; BC, breast cancer

Analyzing immunohistochemistry enabled the evaluation of PTEN protein expression. Normal, weekly, and loss of PTEN expression are illustrated in Figure 1.

**Figure 2:**
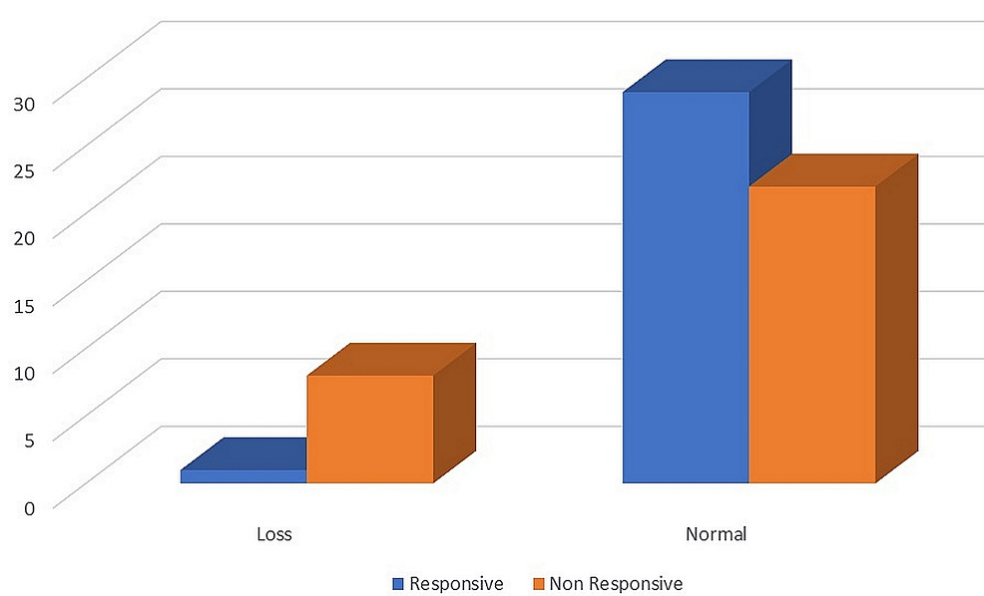
The frequency of PTEN loss in BC women who respond to TRS and those who did not respond PTEN, phosphatase and tensin homolog; BC, breast cancer; TRS, trastuzumab

According to the scoring system of this study, eight specimens (26.67%) among non-responsive women demonstrated PTEN loss compared with one specimen (3.33%) among responsive women. Statistically, there was a significant difference in PTEN loss between responsive and non-responsive women (Figure 2).

Generally, there was no significant association of biochemical markers with PTEN loss (Table 2).

**Table 2:** Association of biochemical markers with PTEN Loss P<0.05 is considered significant. CK-MB, creatine kinase-myocardial band; TnT, troponin T; Tn1, troponin I; PTEN, phosphatase and tensin homolog

Variables	Baseline CK-MB, ng/mL	CK-MB after 12 m, ng/mL	Baseline troponin T, pg/mL	Troponin T after 12 m, pg/mL	Baseline troponin I, pg/mL	Troponin I after 12 m, pg/mL
PTEN present	10.83±2.85	11.54±2.5	134.90±35.23	154.14±60.7	764.9±261.	923.4±265.7
Loss	10.97±2.30	4 11.76±2.22	148.75±142.75	2 139.54±30.82	4 695.9±230.0	825.34±218.8
P-value	0.873	0.785	0.775	0.272	0.419	0.235

## Discussion

At the current time, BC is the most common malignant tumor worldwide, with incidence rates growing every year. There are projected to be 2.3 million new cases in 2020. Because it may be utilized as a genetic target and is important for both diagnosis and treatment, HER2 is a key component of the human epidermal growth factor receptor family [19].

Table 1 lists the demographic data and clinical characteristics of the study's two groups: both the 30 people who did not react and the 30 people who did. Differences between the two groups on a number of metrics are shown statistically in Table 1, along with the related p-values. Neither age nor BMI varies significantly between the non-respondent and respondent groups, as shown by p-values of 0.1. In terms of clinical characteristics, CK-MB, TnT, and Tn1 baseline levels are not significantly different between the two groups (p=0.90, 0.40, and 0.183, respectively). Due to high doses of anthracyclines, notably when combined with TRS, the non-responder group had a higher mean baseline TnI level (1008.93 pg/mL) than the responder group (671.177 pg/mL) after 12 months in the prior trial. Additionally, it has been discovered that elevated cardiac troponin levels in BC patients might predict cardiotoxicity [20]. The cardiac Tn1 and TnT isoforms are very valuable in assessing the risk and diagnosing ACS as well as any other cause, including chemotherapy.

They serve as sensitive and accurate indicators of cardiac injury [21]. There is no evidence to support the use of cardiovascular biomarkers such as NT-pro BNP, CK-MB, or myoglobin to anticipate the onset of cardiovascular issues. The challenge is to treat BC effectively while limiting toxicity [22]. According to the results of the present study, non-responsive women demonstrated a higher frequency of PTEN loss compared with responsive women. These results are in accordance with almost all previous studies in this regard [23,24]. There is evidence from both animal studies and human clinical trials to support this theory. To begin, in vitro and in vivo studies have revealed that PTEN loss may partially reverse TRS's growth-inhibitory effects [25]. Second, poorer clinical outcomes are associated with PTEN loss and/or PIK3CA activating mutations, even when treated with TRS, as shown by multiple retrospective investigations of PTEN in tumors from HER2+ metastatic BC patients [26,27].

In O’Brien et al., 18 different HER2-amplified cell lines were tested for their reactions to TRS and lapatinib [25]. Resistance to TRS was linked to a lack of PTEN or the presence of PI3K-activating mutations, but none of these variables predicted a positive response to lapatinib. An in vitro experiment by Nagata et al. showed that PTEN activation, in addition to its recognized role as a Her2 down-regulator, is one of the most critical pathways that boosts TRS anti-tumor action [28]. By decreasing the amount of Scr bound to the ErbB2 receptor, the author discovered that TRS activates PTEN. This way of testing the TRS function shows that how well it works depends on the level of PTEN, the downregulation of ErbB2, and the stopping of ErbB2-related processes further down the line.

Vogel et al. PTEN loss relates to TRS resistance, and it has been hypothesized that PTEN insufficiency is a biological mechanism that imparts Her2 overexpression in BC [29]. Notably, Yokoyama et al. showed that PTEN loss is linked to a poor response to TRS in people with HER2-gastroesophageal adenocarcinoma; it should not be confused with BC [30]. On the other hand, PTEN levels were checked in a study using the N9831 trial, which included a lot of people with Her2+ BC who were randomly assigned to TRS treatment or no TRS therapy at all, along with combination chemotherapy regimens after surgery. The results of this investigation showed no association between PTEN status and TRS response.

Also, in a study of 129 people with HER2+ cancers that looked back, TRS resistance was not linked to PTEN loss or PI3K mutations. Among them were 48 instances of metastatic BC treated with a combination of taxane and TRS and 26 cases treated with neoadjuvant TRS [28]. This discrepancy between studies could be attributed to several factors, the most important of which are the technique used for the detection of PTEN expression and the cut-off value for considering PTEN loss. Note that a negative result for PTEN staining is best read as being “below the limit of detection of the assay” rather than a definitive lack of PTEN protein. If more sensitive detection technologies are ever developed, this is a crucial consideration to have. Another important point to make is that since immunohistochemistry was used to determine PTEN status in the present study, IHC would overlook any genetic mutation that causes PTEN to lose its function other than a decrease in protein levels. Mutations in the PTEN gene, for instance, are linked to loss of function despite the fact that the PTEN protein is produced [31,32].

The sample size is relatively small, which did not allow for global generalization of the results. Data regarding previous medications for most of the included patients was not available. Thus, serum levels of troponin and MB-CK are not exclusively related to TRS. Rather, other medications could have such an impact. Due to financial and time constraints, an echo study was not used to evaluate the left ventricle ejection fraction, which better reflects the side effects of TRS.

## Conclusions

PTEN loss could be considered a risk factor for TRS resistance in women with Her2+ BC. After 12 months of treatment with TRS, both Tn1 and TnT significantly increased in non-responders compared with responder patients, while CK-MD does not have such an association with TRS response. These findings offer valuable insights into potential factors influencing TRS responsiveness, with the PTEN deletion mutation emerging as a promising candidate for further investigation. Future research and clinical validation studies are warranted to confirm these results and potentially guide treatment decisions for Her2+ BC patients. Understanding the genetic factors associated with treatment response can lead to more personalized and effective therapeutic strategies in BC management.
